# A plasmid module for PCR-based gene modification for the accurate measurement of vacuolar delivery of specific proteins in yeast *Saccharomyces cerevisiae*

**DOI:** 10.1080/27694127.2025.2511724

**Published:** 2025-05-31

**Authors:** Jakob Valdbjørn Kanne, Fulvio Reggiori

**Affiliations:** Department of Biomedicine, Aarhus University, Aarhus C, Denmark

**Keywords:** autophagy, endocytosis, Pho8∆60, turnover, vacuole, vacuolar phosphatase

## Abstract

Monitoring the delivery of single proteins and protein complexes to the vacuole by autophagy or other processes in yeast *Saccharomyces cerevisiae* mainly relies on western blot or fluorescence microscopy analyses using endogenous tagging of the protein of interest with GFP. However, these approaches are semi-quantitative and next to impossible with proteins of low abundancy because of the insensitive nature of the methods. Here, we describe the creation of a new PCR-based integration cassette to endogenously tag specific proteins with the truncated version of the vacuolar phosphatase Pho8. The vacuolar activation of Pho8 allows the quantitative measurement of vacuolar delivery using a colorimetric enzymatic assay. This approach has the advantages of a more quantitative interpretation of data and relies on the appearance of a signal rather than its disappearance. As a proof-of-principle, we examined the vacuolar delivery of known cargoes of bulk autophagy and endocytosis. This new system will be of great value to the whole community working within the field of autophagy and other transport pathways to the vacuole.

## Introduction

Autophagy is a catabolic pathway conserved among eukaryotes and mediated by the autophagy-related (Atg) proteins [1,2]. This process is characterized by the sequestration of proteins, protein complexes and organelles within double-membrane vesicles termed autophagosomes and their delivery into the lysosomes/vacuoles for degradation [1,2]. Autophagy is activated by a plethora of cues, including nutrient starvation [3]. Yeast *S. cerevisiae* is one of the leading organisms for the study of autophagy [4]. There are different assays to monitor bulk autophagy in this model system [5,6], but measuring the vacuolar delivery of specific proteins is more complicated. The current principal strategy to measure the vacuolar delivery of determined proteins or organelles under autophagy-inducing conditions relies mainly on western blots. Here the protein of interest (POI) is tagged with GFP [6–10]. In contrast to most endogenous proteins, GFP is stable in the vacuole and the appearance of free GFP resulting from the proteolysis of the POI fused with it, indicates vacuolar delivery. Thus, the ratio between the free GFP and total GFP (free GFP plus the tagged chimeric protein) provides an estimate of the percentage of protein or organelle delivered into the vacuole [6–10]. This type of approach, however, comes with limitations since western blots are semi quantitative and therefore not optimal to measure minimal and/or exact changes in the delivery dynamics. Protein levels also need to be high as low abundancy proteins are usually difficult to be detected by western blot.

**Figure 1.**
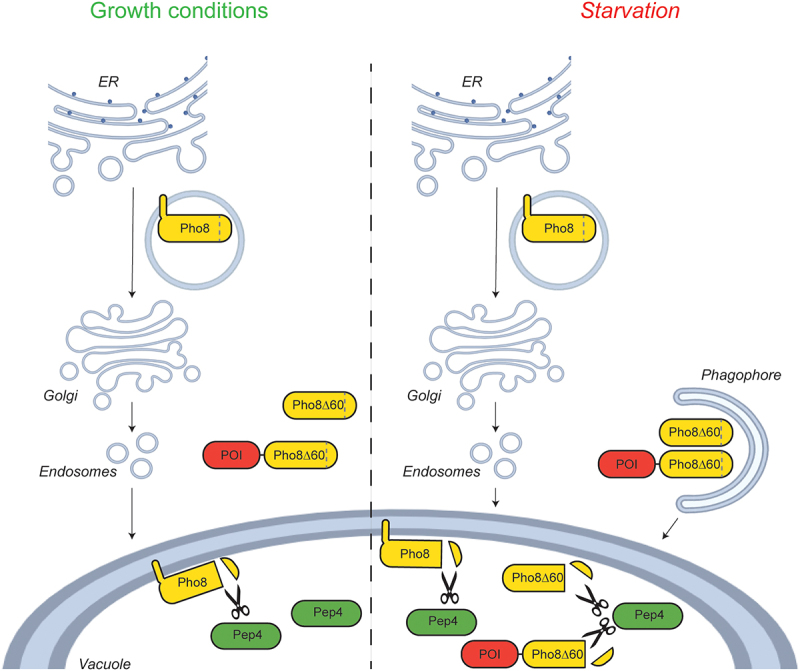
Schematic visualization of the WT Pho8, the Pho8Δ60 truncation routinely used to measure bulk autophagy and the C-terminally PhoΔ60 tagged protein of interest (POI). Under normal growth but also starvation conditions, Pho8 is translated and translocated into the ER from where it is transported to the Golgi and then directly to the vacuole where it is proteolytically activated by Pep4 (the cleavage is highlighted with scissors). The information for Pho8 translocation into ER and transport to the vacuole is contained in its short N-terminal tail and transmembrane domain. Deletion of this part generate a cytoplasmic variant of Pho8 known as PhoΔ60, which can only be transported and activated into the vacuole by bulk autophagy upon autophagy induction by nutrient deprivation [11]. Similarly, a cytoplasmic POI fused with PhoΔ60 can only be transported into the vacuole upon stimulation of bulk autophagy.

A quantitative method used to quantify bulk autophagy is the vacuolar phosphatase (Pho8∆60) assay, which enzymatically measures the vacuolar delivery of Pho8Δ60, a truncated form of Pho8 (phosphate metabolism 8) [6,9,11]. Pho8 is a single transmembrane vacuolar phosphatase that is translocated into the endoplasmic reticulum (ER) and delivered to its final location by passing through part of the secretory and endosomal pathway [12] (Figure 1). Pho8 activity can be quantified *in vitro* with a colorimetric assay, in which the colorless substrate para-nitrophenyl phosphate is dephosphorylated into yellow para-nitrophenol, the absorption of which can be measured photometrically at 400 nm [11]. While its N-terminal transmembrane domain contains the information for insertion into the ER and transport to the vacuole, the C terminus possesses a pro-peptide that is proteolyticly processed in the vacuole by the Pep4 (carboxypeptidase Y-deficient 4) protease, leading to Pho8 activation [12]. Truncation of the first 60 amino acids of Pho8 generates Pho8Δ60, a cytoplasmic variant that is still in its inactive form (Figure 1). Pho8Δ60 can only be transported to and activated in the vacuole by bulk autophagy [11]. Thus, in a strain background that lacks endogenous *PHO8*, the progression of bulk autophagy can be quantified by measuring the vacuolar delivery by colorimetrically assessing the activity of this enzyme [11]. Deletion of *PHO13* can further reduce the phosphatase background activity making the enzymatic assay even more sensitive [6]. This strategy has also been exploited to assess the selective turnover of mitochondria and the ER, i.e., mitophagy and reticulophagy/ER-phagy, by fusing Pho8Δ60 with a mitochondrial targeting signal or endogenous ER membrane spanning peptides, respectively [13,14]. Similarly, Pho8Δ60 has also been fused with misfolded transmembrane proteins to measure their vacuolar delivery via the endosomal system [15].

To overcome the current limitations in measuring the autophagic delivery into the vacuole of single proteins and organelles, we decided to generate a plasmid cassette to endogenously fuse individual POIs with Pho8Δ60 through PCR-based recombination. We opted for this strategy since it presents two major advantages in comparison to endogenous tagging with GFP. First, it facilitates the detection of low expressed proteins since it would be sufficient to prolong the time of the enzymatic measurement. Second, the Pho8Δ60 assay gives more precise quantitative datasets that, because of the sensitivity of an enzymatic assay in comparison to western blots, allows the detection of small differences between samples. Indeed, we show that the new plasmid cassette allows measuring the vacuolar delivery of endogenous proteins by either autophagy or endocytosis.

## Results

### The Pho8Δ60 tag can be functionally fused to endogenous proteins allowing the measurement of their vacuolar delivery

The pFA6a plasmid cassette is a widely used system for the PCR-based chromosomal integration of tags at the 3’ of genes through homologues recombination, which results in the endogenous expression of proteins fused at their C terminus with a tag of choice [16]. Practically, the PCR primers used have at their 5’ ends that correspond to the desired target gene sequences, i.e., the last 40–60 nucleotides of the open reading frame (excluding the stop codon) and the first 40–60 nucleotides in the terminator, and at their 3’ ends approximately 20 nucleotides that anneal to and allow amplification of the sequence encoding a tag and the selectable marker gene in the template plasmid [16]. The amplified DNA is directly transformed into yeast and homologous recombinants are identified on selection plates appropriate for the employed selectable marker. The correctness of the insertion is then verified by DNA sequencing or western blot. We decided to take advantage of the pFA6a system to develop our tagging cassette for the measurement of vacuolar delivery. Thus, we amplified the *Pho8Δ60* sequence by PCR and cloned it into the pFA6a-GFP-natMX6 plasmid to replace GFP (see Materials and Methods for the details). The resulting pFA6a- Pho8Δ60-natMX6 plasmid, which carries the nourseothricin resistance selectable marker, was then used to generate the strains for the successive testing.

**Figure 2.**
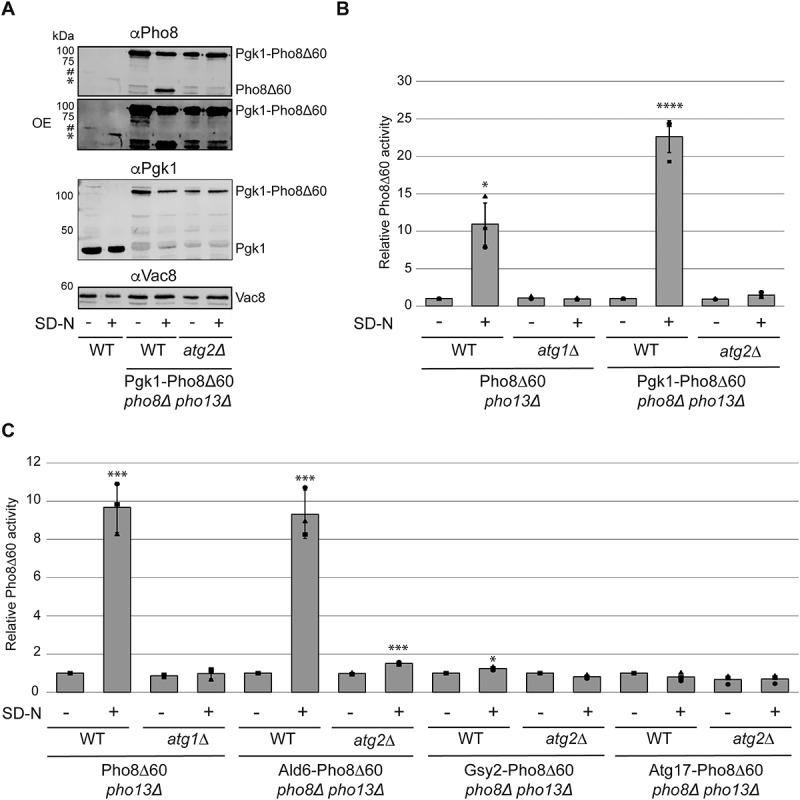
Pho8Δ60 can be endogenously fused to proteins through DNA homologous recombination and functions as a proxy for vacuolar delivery. (A) WT cells carrying endogenous Pho8 (SEY6210) or the *pho8∆ pho13∆* strain expressing Pgk1-Pho8Δ60 (JKY005) and lacking *ATG2* (JKY006) were grown in YPD to log phase and nitrogen starved in SD-N for 4 h. Protein extracts from cells before and after nitrogen starvation were separated by individual gels and examined by western blot using anti-Pgk1, anti-Pho8 and anti-Vac8 antibodies. An overexposed (OE) anti-Pho8 blot is also provided to better show endogenous Pho8, the levels of which are lower than those of the Pgk1-Pho8Δ60 chimera as it is expressed by the *PHO8* promoter and not the strong *PGK1* promoter. Abundant Vac8 was used as the loading control instead of Pgk1, a standard loading control, since tagged in some of the strains examined in this experiment. The symbol # indicates precursor Pho8, while * highlights proteolytically cleaved, mature Pho8. (B) The *pho8∆ pho13∆* or *atg1∆ pho8∆ pho13∆* cells carrying Pho8Δ60 (WLY176 and RGY352, respectively), or the *pho8∆ pho13∆* mutant expressing Pgk1-Pho8Δ60 (JKY005) with or without *ATG2* (JKY006) were grown as in panel B. The Pho8Δ60 assay was carried out with extracts from cells collected before and after the 4 h of nitrogen starvation. Bars represent the average activity relative to the WT grown in rich medium (YPD) from three different experiments plus standard deviation (SD). (C) The *pho8∆ pho13∆* or *atg1∆ pho8∆ pho13∆* cells carrying Pho8Δ60 (WLY176 and RGY352, respectively), or the *pho8∆ pho13∆* or *atg2∆ pho8∆ pho13∆* strain expressing Ald6-Pho8Δ60 (JKY026 and JKY043), Gsy2-Pho8Δ60 (JKY042 and JKY046) or Atg17-Pho8Δ60 (JKY056 and JKY057) respectively, were grown and analyzed as in Figure 1B. The Pho8Δ60 assay was carried out with extracts from cells collected before and after the 4 h of nitrogen starvation. Bars represent the average activity relative to the WT grown in rich medium (YPD) from three different experiments. plus SD. *p ≤0.05, ***p ≤0.001, ****p ≤0.0001.

We then assessed the functionality of the Pho8Δ60 cassette by endogenously tagging Pgk1 (phosphoglycerate kinase 1), a protein often used as a proxy to measure bulk autophagy progression using the GFP-based processing assay [10]. We first examined the processing of the Pgk1-Pho8Δ60 chimera in the vacuole by western blot in the *pho8∆ pho13∆* and the autophagy-deficient *atg2∆ pho8∆ pho13∆* strain, which lacks the lipid transferase essential for autophagosome formation [17,18]. As expected, the anti-Pho8 and anti-Pgk1 antibodies recognized bands corresponding to endogenous Pho8 and Pgk1 in WT cells in both growing and starvation conditions (Figure 2A). A size shift of endogenous Pho8 after nitrogen starvation was observed, reflecting the increased production of the mature protein (Figure 2A) [10]. Importantly, we detected a band consistent with the predicted molecular weight of the Pgk1-Pho8Δ60 chimera with both the anti-Pgk1 and anti-Pho8 antibody in both *pho8∆ pho13∆* and *atg2∆ pho8∆ pho13∆* cells expressing this fusion protein (Figure 2A).

Next, we tested the functionality of the Pho8Δ60 tag by nitrogen starving the *pho8∆ pho13∆* and *atg2∆ pho8∆ pho13∆* strains expressing Pgk1-Pho8Δ60 for 4 h to induce bulk autophagy and measuring the phosphatase activity in cell extracts. As controls, we employed *pho8∆ pho13∆* and the autophagy-deficient *atg1∆ pho8∆ pho13∆* cells, which lacks the protein kinase subunit of the Atg1 complex and thus they display a block in bulk autophagy [19], carrying cytosolic Pho8Δ60, which are routinely used as the positive and negative control in experiments assessing bulk autophagy progression with the Pho8Δ60 assay [11]. Pho8Δ60 was activated in the vacuole upon nitrogen starvation in *pho8∆ pho13∆* cells but not in the *atg1∆ pho8∆ pho13∆* strain, showing that Pho8Δ60 is delivered into the vacuole by autophagy as expected (Figure 2B). Importantly, we detected the Pho8Δ60 signal in *pho8∆ pho13∆* cells expressing Pgk1-Pho8Δ60 upon autophagy induction by nitrogen starvation but not in the *atg2∆ pho8∆ pho13∆* mutant (Figure 2B). This result confirmed that Pgk1-Pho8Δ60 is delivered to the vacuole by autophagy.

Next, we set out to validate the system using additional positive and negative controls, we endogenously tagged the cytoplasmic aldehyde dehydrogenase Ald6 (aldehyde dehydrogenase 6), another established autophagosomal cargo protein under nitrogen starvation conditions, the cytoplasmic enzyme Gsy2 (glycogen synthase 2), as well as Atg17, which have been found to not be an autophagy target under the same conditions [20–23]. The *pho8∆ pho13∆* and *atg2∆ pho8∆ pho13∆* strains expressing Ald6-Pho8Δ60, Gsy2-Pho8Δ60 or Atg17-Pho8Δ60 were transferred to a medium lacking nitrogen and Pho8Δ60 activity was measured before and after this nutritional shift (Figure 2C). As controls, we again used *pho8∆ pho13∆* and *atg1∆ pho8∆ pho13∆* cells carrying cytosolic Pho8Δ60. This analysis revealed a transport of the Ald6-Pho8Δ60 fusion into the vacuole in an autophagy-dependent manner (Figure 2C). In contrast, the Gsy2-Pho8Δ60 and the Atg17-Pho8Δ60 fusions showed no vacuolar delivery after nitrogen starvation (Figure 2C). These results further cemented the Pho8Δ60 tag as a specific proxy readout for vacuolar delivery of proteins and showed that the tag itself does not promote vacuolar delivery. To increase its dexterity in strain building, we replaced the initial selectable marker natMX with an array of selectable markers for antibiotics or amino acids (Fig. S1 and Table 1).

**Table 1.** Plasmids Generated in This Study.

Plasmid name	Selectable marker	PCR product size [kb]
pFA6a-Pho8Δ60-HIS3MX6	*HIS3*	1,569
pFA6a-Pho8Δ60-K.l.LEU2MX6	*Kluyveromyces lactis LEU2*	2,508
pFA6a-Pho8Δ60-TRP1	*TRP1*	1,159
pFA6a-Pho8Δ60-C.a.URA3MX6	*Candida albicans URA3*	1,702
pFA6a-Pho8Δ60-bleX6	phleomycin resistance*	1,093
pFA6a-Pho8Δ60-hphNT1	hygromycin resistance*	1,679
pFA6a-Pho8Δ60-natMX6	nourseothricin resistance*	1,293

*Antibiotics were used at the following concentrations in this study: 200 µg/ml phleomycin/zeocin, 300 µg/ml hygromycin, and 100 µg/ml nourseothricin. Of note, the minimum inhibitory concentration of phleomycin must be determined for the used strain background before employing it for selection, because it can vary.

## The Pho8Δ60 tagging system allows the measurement of endocytosis

**Figure 3.**
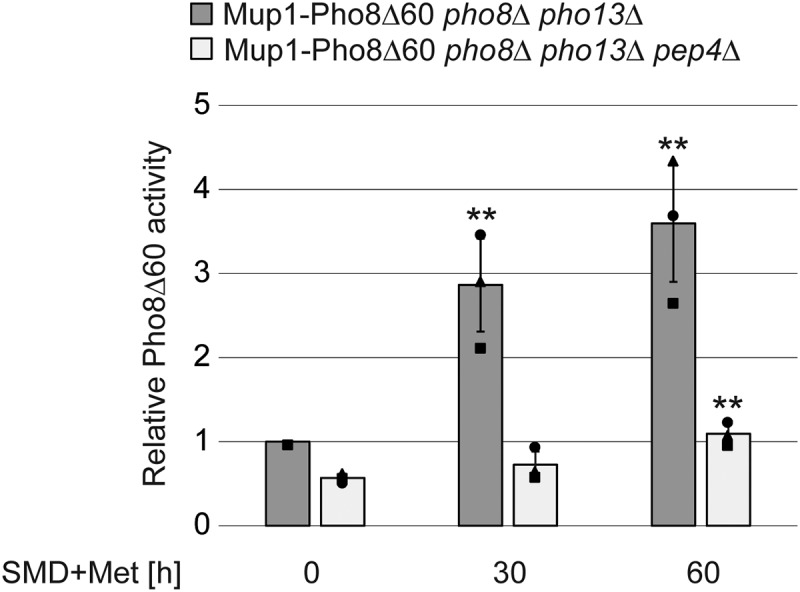
Pho8Δ60 can be endogenously fused to Mup1 through DNA homologous recombination and functions as a proxy for endocytosis. (A) The *pho8∆ pho13∆* or *pep4∆ pho8∆ pho13∆* cells expressing the Mup1-Pho8Δ60 fusion (JKY044 and JKY047) were grown in SMD without methionine to log phase and shifted to SMD containing 20 mg/l methionine for 60 min. The phosphatase assay was carried out with extracts from cells collected after 0, 30 and 60 min of medium shift. Bars represent the average activity relative to the WT at time 0 from three different experiments plus SD. **p ≤0.01.

The turnover of plasma membrane components relies on endocytosis, a process in which portions of the plasma membrane invaginate, forming vesicles that in yeast are transported to a unique comportment that has characteristics of the mammalian trans-Golgi network and both early and recycling endosomes [24]. This compartment then matures into late endosomes, which finally fuse with lysosomes/vacuoles, delivering the endocytosed material in the interior of these organelles for degradation [24]. In yeast, endocytosis and transport through these organelles can be followed by monitoring the trafficking of methionine transporter Mup1 (methionine uptake 1) from the plasma membrane to the vacuole upon withdrawal of methionine from the growth medium (e.g [25]). This analysis is routinely carried out by tagging Mup1 with GFP and imaging this chimera by fluorescence microscopy (e.g [26]). We opted to exploit Mup1 for a proof of concept to extend the C-terminal tagging with Pho8Δ60 to the analysis of the vacuolar transport of endocytosed proteins. Pho8Δ60 tagging has previously been used to assess the vacuolar degradation of misfolded transmembrane proteins delivered from the Golgi to the endosomal system [15]. Mup1 was thus tagged in the *pho8∆ pho13∆* strain but also in the same cells lacking *PEP4*, to inhibit the Pho8Δ60 activation since endocytosis functions independently of the core autophagy machinery and consequently deletion of an *ATG* gene cannot be used as a control [27]. When we measured the Pho8Δ60 activity in *pho8∆ pho13∆* cells expressing Mup1-Pho8Δ60 upon transfer to a medium containing methionine for 0, 30, and 60 min, we observed a gradual increase of the signal indicative of the endocytosis and vacuolar delivery of Mup1 (Figure 3). This signal was absent in the *pep4∆ pho8∆ pho13∆* strain showing the specificity of the assay (Figure 3). This result shows that the Pho8Δ60 tag can also be used to investigate transport to the vacuole via the endosomal system.

## Discussion

The currently most used standard method to assess the vacuolar delivery of specific proteins or organelles relies on the tagging of the POI with GFP and visualization of the band corresponding to free GFP generated upon delivery into the vacuole by western blot [6–10]. Here, we describe a new approach that makes use of the Pho8Δ60 reporter (Figure 1). This system provides some major advantages over the GFP processing assays in measuring vacuolar delivery. The first is that the changes in Pho8∆60 activity, especially when minor, can be more precisely quantified than differences in the levels of free GFP by western blot. The second major advantage is that while low abundance proteins cannot be detected by anti-GFP western blots, Pho8∆60 activity allows to carry out the enzymatic activity as long as it is sufficient to detect a clear signal and thus assess differences. Here we were able to easily monitor abundant proteins such as Pgk1 (535,389 copies per cell; Saccharomyces Genome Database, SGD, https://www.yeastgenome.org/) and Ald6 (135,000 copies per cell) as well as ones expressed at low levels such as Gsy2 (4,748 copies per cell), Mup1 (4,102 copies per cell), and Atg17 (746 copies per cell). A connected benefit is that Pho8∆60 fusion proteins with diverse expression levels can be examined on the same 96-wells plate as signals with different intensity in adjacent wells do not cause problems in detection, which is not the case in western blot analyses without prior adjustments to load the same amount of each GFP-tagged proteins [23]. Third, our plasmid cassette is based on the pFA6a system [16], which is employed by a multitude of yeast laboratories to endogenously fuse proteins with numerous different tags. As a result, the same set of primers for the PCR amplification used in the pFA6a system is compatible with our cassette. Finally, another advantage when using the Pho8Δ60 as a reporter is the large number of samples that can be processed in parallel, allowing high-throughput screens, since the Pho8Δ60 assay is compatible with a 96-well-plate format [6].

A possible disadvantage when using Pho8Δ60 is its size of 60 kDa; this is larger than GFP, which is approximately 27 kDa. Large tags, but also simply the tagging of the C terminus of the POI, can interfere in specific cases with the function of the protein. A possible strategy to circumvent this problem is to tag the N terminus of the POI. Another consideration to be kept in mind is that when assessing the delivery of an integral vacuole membrane protein, Pho8Δ60 needs to be fused with the terminus of the POI that is oriented into the vacuolar lumen to be accessible for the proteolytic activation by Pep4.

In summary, we have created a modular tool to precisely measure vacuolar delivery of POIs using Pho8Δ60 as a proxy tag that circumvents some limitations of the current GFP cleavage approaches. We believe that this new plasmid cassette will be a new very valuable experimental tool for the study of autophagy and endosomal transport.

## Materials and methods

### Plasmids

The plasmids in Fig. S1 and Table 1 were created by first amplifying the *PHO8Δ60* sequence by PCR from the pPHO8D60-PSSO416 plasmid [15] and replacing the *GFP* sequence in the pFA6a-GFP-natMX6 vector [28] using PacI and AscI. The resulting pFA6a-Pho8Δ60-natMX6 plasmid was also used as the backbone into which the different selectable markers were subsequently cloned upon removing the *natMX6* gene using AscI/EcoRI or AscI/EcoRV. The introduced selectable makers were excised from the pFA6a-GFP (S65T)-hphNT1 (AscI/EcoRV) [28], pFA6a-Pho8Δ60-bleMX6 (AscI/EcorV) [29], pFA6a-GFP (S65T)-TRP1 (AscI/EcoRI) [16], pFA6a-GFP (S65T)-HIS3MX6 (AscI/EcoRI) [16], pUG72 (*C.a*.URA3) (AscI/EcoRV) [30], and pUG73 (*K.l*.LEU2) (AscI/EcoRI) [30] plasmids. This generated the pFA6a-Pho8Δ60-hphNT1, pFA6a-Pho8Δ60-TRP1MX6, pFA6a-Pho8Δ60-HISMX6, pFA6a-Pho8Δ60-bleMX6, pFA6a-Pho8Δ60-C.a.URA3MX6, and pFA6a-Pho8Δ60-K.I.LEU2MX6 plasmid, respectively (Fig. S1 and Table 1).

## Yeast strains and media

All *S. cerevisiae* strains used in this study were created in the haploid SEY6210 background [31] and they are listed in Table 2. For gene deletion, the *PEP4 or ATG2* coding regions were replaced through PCR-based recombination with the *kanamycin-resistance gene* flanked by *loxP* sites *(loxP-kanMX-loxP)* using primers containing 60 bases of identity to the regions flanking the open reading frame [16]. The PCR products were transformed into yeast using a standard lithium acetate protocol [33]. Gene knockouts were verified by polymerase chain reaction (PCR) and/or precursor Ape1 (aminopeptidase I) processing analysis. PCR-based integration of *Pho8∆60* the 3’ end of *PGK1, ALD6, GSY2, ATG17,* and *MUP1* was used to generate strains expressing C-terminal fusion proteins under the control of the authentic promoters. The plasmid template for integration was pFA6a-Pho8∆60-natMX. Correct integration was examined by PCR and expression of tagged proteins verified by western blot.

**Table 2.** Strains Used in This Study.

Name	Genotype	Reference
FRY031	SEY6210 *pho8Δ pho13Δ*	[15]
JKY005	SEY6210 *pho8Δ pho13Δ PGK1-PHO8∆60:natMX*	This study
JKY006	SEY6210 *pho8Δ pho13Δ PGK1-PHO8∆60:natMX atg2Δ::kanMX*	This study
JKY042	SEY6210 *pho8Δ pho13Δ GSY2-PHO8∆60:natMX*	This study
JKY046	SEY6210 *pho8Δ pho13Δ GSY2-PHO8∆60:natMX atg2Δ::kanMX*	This study
JKY026	SEY6210 *pho8Δ pho13Δ ALD6-PHO8∆60:natMX*	This study
JKY043	SEY6210 *pho8Δ pho13Δ ALD6-PHO8∆60:natMX atg2Δ::kanMX*	This study
JKY044	SEY6210 *pho8Δ pho13Δ MUP1-PHO8∆60:natMX*	This study
JKY047	SEY6210 *pho8Δ pho13Δ MUP1-PHO8∆60:natMX/pep4:kanMX*	This study
RGY352	SEY6210 *PHO8∆60 pho13∆ atg1Δ::hphNT1*	This study
JKY056	SEY6210 *pho8Δ pho13Δ ATG17-PHO8∆60:natMX*	This study
JKY057	SEY6210 *pho8Δ pho13Δ ATG17-PHO8∆60:natMX atg2Δ::kanMX*	This study
SEY6210	*MATα leu2–3,112 ura3–52 his3-∆200 trp1-∆901 lys2–801 suc2-∆9 GAL*	[31]
WLY176	SEY6210 *PHO8∆60 pho13∆*	[32]

Yeast cells were grown in rich medium (YPD; 1% yeast extract, 2% peptone, 2% glucose) or synthetic minimal medium (SMD; 0.69% yeast nitrogen base, 1% glucose, vitamins, and amino acids including or not 20 mg/l methionine), and nitrogen starved in a synthetic medium lacking nitrogen (SD-N; 0.17% yeast nitrogen base without amino acids, 2% glucose). All medium components were from Formedium, Swaffman, the United Kingdom.

## Western blotting

For western blot analyses, 2.5 OD_600_ equivalent units were collected by centrifugation at 13,000 *g* for 1 min and resuspended in 500 μl of ice-cold 10% trichloroacetic acid. After leaving them on ice for 30 min, samples were centrifuged at 13,000 × *g* for 5 min at 4°C and protein pellets were resuspended in 1 ml of ice-cold acetone by sonication. Samples were then stored at −20°C for at least 30 min before being centrifuged at 13,000 × *g* for 5 min at 4°C. After removal of the acetone and drying, the protein pellets were resuspended in 80 μl of Laemmli sample buffer (2% SDS, 8.7% glycerol, 0.08 M Tris-HCl, pH 6.8, 2.5% 2-mercaptoethanol, 0.002% bromophenol blue) and boiled before loading the samples on SDS-PAGE gels. Western blot membranes were probed with a mouse anti-Pho8 antibody (1:500 dilution; Abcam, ab113688), or anti-Pgk1 (1:7500 dilution) [34] and/or anti-Vac8 (vacuole-related 8; 1:1000 dilution) [35] rabbit antisera. Secondary antibodies were Alexa Fluor® 680 conjugated goat anti-rabbit (1:7500 dilution; Invitrogen, A-21109) or anti-mouse IgG (1:7500 dilution; Invitrogen, A-21058). Detection of proteins and quantification of non-saturated images were performed using an Odyssey® Fc Imaging System (LI-COR Biosciences, Lincoln, NE).

## Pho8∆60 assay

This assay to measure the bulk autophagy was conducted as previously described [6]. Five OD_600_ equivalents of cells were harvested at the analyzed time points by centrifugation at 3,000 rpm for 5 min before and after starvation. The cells were then lysed in 200 μl of the lysis buffer (20 mm PIPES, pH 6.8, 0.5% Triton X-100 [Sigma Aldrich, X100-500 ml], 50 mm KCl, 100 mm potassium acetate, 10 mm MgCl_2_, 10 µM ZnSO_4_, 2 mm PMSF [Sigma Aldrich, P7626-5 G]) by adding 100 μl of glass beads (0.4–0.6 mm in diameter; VWR, 412–0069) and vortexing at 4°C for 5 min. Lysates were then centrifuged at 15,000 × g for 5 min at 4°C. 50 μl of supernatant were mixed with 200 μl of Pho8Δ60 phosphatase reaction buffer (250 mm Tris-HCl, pH 8.5, 0.4% Triton X-100, 10 mm MgCl_2_, 10 µM ZnSO_4_, and freshly added 1.25 mm *p*-nitrophenyl phosphate [Sigma Aldrich, N2765]) prewarmed at 37°C in 96-well plates and the solution color was measured at 405 nm and 37°C using GloMax® Discover Microplate Reader (Promega, Madison, WI, USA) at 1-min intervals during 40 min. The protein concentration in the samples was determined using the BCA protein assay kit (Thermo Fisher Scientific 23,227) and the GloMax® Discover Microplate Reader following the manufacturer instructions. The Pho8∆60 activity was calculated as following: The absorbance at the time points of linear growth was used to draw a linear plot for each sample before to divide the slope of the plot by the amount of protein. The resulting activity in arbitrary units was expressed relative to the Pho8∆60 activity of WT cells, which was set to 1. Statistical analyses were carried using the two tailed unpaired t-test and significant differences (*p*) are indicated in the graphs.

## Supplementary Material

Figure S1.tif

## Data Availability

The data that support the findings of this study are available from the corresponding author upon reasonable request.

## Abbreviations

Ald6: aldehyde dehydrogenase 6
Ape1: aminopeptidase I
Atg: autophagy related
ER: endoplasmic reticulum
GFP: green fluorescent protein
Gsy2: glycogen synthase 2
Mup1: methionine uptake 1
Pep4: carboxypeptidase Y-deficient 4
Pgk1: phosphoglycerate kinase 1
Pho8: phosphate metabolism 8
Pho13: phosphate metabolism 13
POI: protein of interest
Vac8: vacuole-related 8

## References

[1] Gomez-Sanchez R, Tooze SA, Reggiori F. Membrane supply and remodeling during autophagosome biogenesis. Curr Opin Cell Biol. 2021;71:112–15. doi: 10.1016/j.ceb.2021.02.001 33930785

[2] Hu Y, Reggiori F. Molecular regulation of autophagosome formation. Biochem Soc Trans. 2022;50(1):55–69. doi: 10.1042/BST20210819 35076688 PMC9022990

[3] Lahiri V, Hawkins WD, Klionsky DJ. Watch what you (self-) eat: autophagic mechanisms that modulate metabolism. Cell Metab. 2019;29(4):803–826. doi: 10.1016/j.cmet.2019.03.003 30943392 PMC6450419

[4] Reggiori F, Klionsky DJ. Autophagic processes in yeast: mechanism, machinery and regulation. Genetics. 2013;194(2):341–361. doi: 10.1534/genetics.112.149013 23733851 PMC3664846

[5] Gomez-Sanchez R, Sanchez-Wandelmer J, Reggiori F. Monitoring the formation of autophagosomal precursor structures in yeast *Saccharomyces cerevisiae*. Methods Enzymol. 2017;588:323–365. 28237109 10.1016/bs.mie.2016.09.085

[6] Guimaraes RS, Delorme-Axford E, Klionsky DJ, et al. Assays for the biochemical and ultrastructural measurement of selective and nonselective types of autophagy in the yeast *Saccharomyces cerevisiae*. Methods. 2015;75:141–150. doi: 10.1016/j.ymeth.2014.11.023 25484341

[7] Shintani T, Klionsky DJ. Cargo proteins facilitate the formation of transport vesicles in the cytoplasm to vacuole targeting pathway. J Biol Chem. 2004;279(29):29889–29894. doi: 10.1074/jbc.M404399200 15138258 PMC1712665

[8] Cheong H, Klionsky DJ. Biochemical methods to monitor autophagy-related processes in yeast. Methods Enzymol. 2008;451:1–26. 19185709 10.1016/S0076-6879(08)03201-1

[9] Torggler R, Papinski D, Kraft C. Assays to monitor autophagy in *Saccharomyces cerevisiae*. Cells. 2017;6(3):23. doi: 10.3390/cells6030023 28703742 PMC5617969

[10] Welter E, Thumm M, Krick R. Quantification of nonselective bulk autophagy in *S. cerevisiae* using Pgk1-GFP. Autophagy. 2010;6(6):794–797. doi: 10.4161/auto.6.6.12348 20523132

[11] Noda T, Matsuura A, Wada Y, et al. Novel system for monitoring autophagy in the yeast *Saccharomyces cerevisiae*. Biochem Biophys Res Commun. 1995;210(1):126–132. doi: 10.1006/bbrc.1995.1636 7741731

[12] Klionsky DJ, Emr SD. Membrane protein sorting: biosynthesis, transport and processing of yeast vacuolar alkaline phosphatase. Embo J. 1989;8(8):2241–2250. doi: 10.1002/j.1460-2075.1989.tb08348.x 2676517 PMC401154

[13] Campbell CL, Thorsness PE. Escape of mitochondrial DNA to the nucleus in ymel yeast is mediated by vacuolar-dependent turnover of abnormal mitochondrial compartments. J Cell Sci. 1998;111(16):2455–2464. doi: 10.1242/jcs.111.16.2455 9683639

[14] Schuck S, Gallagher CM, Walter P. ER-phagy mediates selective degradation of endoplasmic reticulum independently of the core autophagy machinery. J Cell Sci. 2014;127:4078–4088. doi: 10.1242/jcs.154716 25052096 PMC4163648

[15] Reggiori F, Black MW, Pelham HRB, et al. Polar transmembrane domains target proteins to the interior of the yeast vacuole. Mol Biol Cell. 2000;11(11):3737–3749. doi: 10.1091/mbc.11.11.3737 11071903 PMC15033

[16] Longtine MS, McKenzie A, Demarini DJ, et al. Additional modules for versatile and economical PCR-based gene deletion and modification in *Saccharomyces cerevisiae*. Yeast. 1998;14(10):953–961. doi: 10.1002/(SICI)1097-0061(199807)14:10<953::AID-YEA293>3.0.CO;2-U 9717241

[17] Osawa T, Kotani T, Kawaoka T, et al. Atg2 mediates direct lipid transfer between membranes for autophagosome formation. Nat Struct Mol Biol. 2019;26(4):281–288. doi: 10.1038/s41594-019-0203-4 30911189

[18] Valverde DP, Yu S, Boggavarapu V, et al. ATG2 transports lipids to promote autophagosome biogenesis. J Cell Biol. 2019;218(6):1787–1798. doi: 10.1083/jcb.201811139 30952800 PMC6548141

[19] Kabeya Y, Kamada Y, Baba M, et al. Atg17 functions in cooperation with Atg1 and Atg13 in yeast autophagy. Mol Biol Cell. 2005;16(5):2544–2553. doi: 10.1091/mbc.e04-08-0669 15743910 PMC1087256

[20] Takeda E, Isoda T, Hosokawa S, et al. Receptor-mediated cargo hitchhiking on bulk autophagy. Embo J. 2024;43(15):3116–3140. doi: 10.1038/s44318-024-00091-8 38755257 PMC11294605

[21] Suzuki K, Nakamura S, Morimoto M, et al. Proteomic profiling of autophagosome cargo in *Saccharomyces cerevisiae*. PLOS ONE. 2014;9(3):e91651. doi: 10.1371/journal.pone.0091651 24626240 PMC3953483

[22] Onodera J, Ohsumi Y. Ald6p is a preferred target for autophagy in yeast, *Saccharomyces cerevisiae*. J Biol Chem. 2004;279(16):16071–16076. doi: 10.1074/jbc.M312706200 14761979

[23] Isoda T, Takeda E, Hosokawa S, et al. Atg45 is an autophagy receptor for glycogen, a non-preferred cargo of bulk autophagy in yeast. iScience. 2024;27(6):109810. doi: 10.1016/j.isci.2024.109810 38832010 PMC11145338

[24] Day KJ, Casler JC, Glick BS. Budding yeast has a minimal endomembrane system. Dev Cell. 2018;44(1):56–72.e4. doi: 10.1016/j.devcel.2017.12.014 29316441 PMC5765772

[25] Lin CH, MacGurn JA, Chu T, et al. Arrestin-related ubiquitin-ligase adaptors regulate endocytosis and protein turnover at the cell surface. Cell. 2008;135(4):714–725. doi: 10.1016/j.cell.2008.09.025 18976803

[26] Guiney EL, Klecker T, Emr SD, et al. Identification of the endocytic sorting signal recognized by the Art1–Rsp5 ubiquitin ligase complex. Mol Biol Cell. 2016;27(25):4043–4054. doi: 10.1091/mbc.E16-08-0570 27798240 PMC5156545

[27] Li SC, Kane PM. The yeast lysosome-like vacuole: endpoint and crossroads. Biochim Biophys Acta. 2009;1793(4):650–663. doi: 10.1016/j.bbamcr.2008.08.003 18786576 PMC2906225

[28] Goldstein AL, McCusker JH. Three new dominant drug resistance cassettes for gene disruption in *Saccharomyces cerevisiae*. Yeast. 1999;15(14):1541–1553. doi: 10.1002/(SICI)1097-0061(199910)15:14<1541::AID-YEA476>3.0.CO;2-K 10514571

[29] Drocourt D, Calmels T, Reynes JP, et al. Cassettes of the *Streptoalloteichus hindustanus* ble gene for transformation of lower and higher eukaryotes to phleomycin resistance. Nucleic Acids Res. 1990;18(13):4009. doi: 10.1093/nar/18.13.4009 1695734 PMC331125

[30] Gueldener U, Heinisch, J, Koehler, GJ, et al. A second set of *loxP* marker cassettes for cre-mediated multiple gene knockouts in budding yeast. Nucleic Acids Res. 2002;30(6):23e–23. doi: 10.1093/nar/30.6.e23 PMC101367 11884642

[31] Robinson JS, Klionsky DJ, Banta LM, et al. Protein sorting in *Saccharomyces cerevisiae*: isolation of mutants defective in the delivery and processing of multiple vacuolar hydrolases. Mol Cell Biol. 1988;8(11):4936–4948. doi: 10.1128/MCB.8.11.4936 3062374 PMC365587

[32] Mao K, Chew LH, Inoue-Aono Y, et al. Atg29 phosphorylation regulates coordination of the Atg17-Atg31-Atg29 complex with the Atg11 scaffold during autophagy initiation. Proc Natl Acad Sci USA. 2013;110(31):E2875–84. doi: 10.1073/pnas.1300064110 23858448 PMC3732952

[33] Gietz RD, Woods RA. Yeast transformation by the LiAc/SS carrier DNA/PEG method. Methods Mol Biol. 2006;313:107–120. 16118429 10.1385/1-59259-958-3:107

[34] Sanchez-Wandelmer J, Kriegenburg F, Rohringer S, et al. Atg4 proteolytic activity can be inhibited by Atg1 phosphorylation. Nat Commun. 2017;8(1):295. doi: 10.1038/s41467-017-00302-3 28821724 PMC5562703

[35] Veit M, Laage R, Dietrich L, et al. Vac8p release from the SNARE complex and its palmitoylation are coupled and essential for vacuole fusion. Embo J. 2001;20(12):3145–3155. doi: 10.1093/emboj/20.12.3145 11406591 PMC150195

