# Supplementary figures and images for: A plasmid module for PCR-based gene modification for the accurate measurement of vacuolar delivery of specific proteins in yeast Saccharomyces cerevisiae
Source: Autophagy Rep. 2025 May 31;4(1):2511724. doi: 10.1080/27694127.2025.2511724 (PMC12128659; doi:10.1080/27694127.2025.2511724)

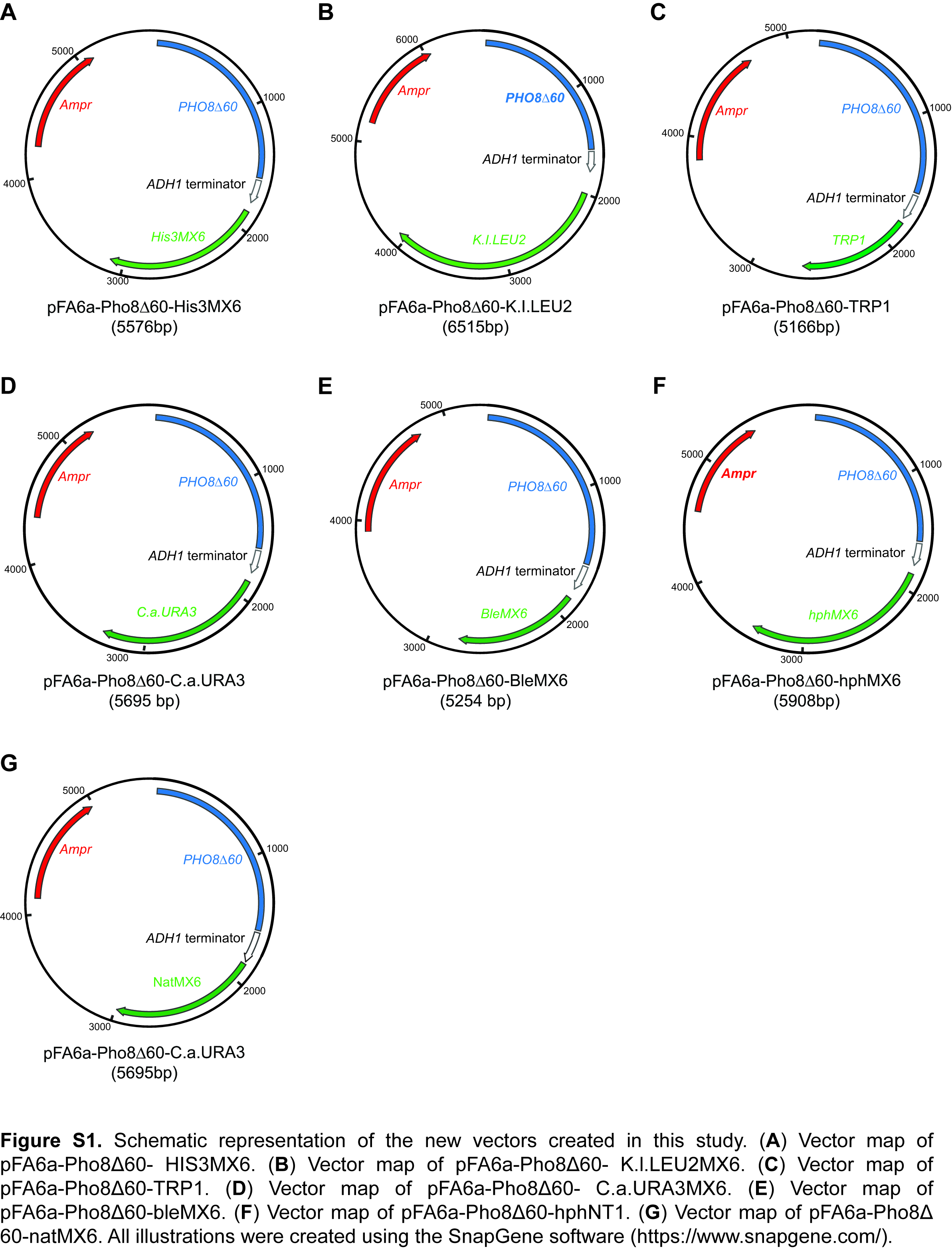

Supplement: Figure S1.tif [file KAUO_A_2511724_SM2974.tif]
